# Filtered Saliva for Rapid and Accurate Analyte Detection for POC Diagnostics

**DOI:** 10.3390/diagnostics14111088

**Published:** 2024-05-24

**Authors:** Nadia Farsaeivahid, Christian Grenier, Ming L. Wang

**Affiliations:** 1 Interdisciplinary Engineering Program, College of Engineering, Northeastern University, Boston, MA 02115, USA; farsaeivahid.n@northeastern.edu (N.F.); grenier.ch@northeastern.edu (C.G.); 2 Civil and Environmental Engineering Department, Northeastern University, Boston, MA 02115, USA

**Keywords:** diagnostics, saliva, electrochemical biosensor, point of care (POC) devices, glucose, SARS-CoV-2 spike protein, saliva filtration process

## Abstract

Saliva has shown considerable promise as a diagnostic medium for point-of-care (POC) and over-the-counter (OTC) diagnostic devices due to the non-invasive nature of its collection. However, a significant limitation of saliva-based detection is undesirable interference in a sensor’s readout caused by interfering components in saliva. In this study, we develop standardized sample treatment procedures to eliminate bubbles and interfering molecules while preserving the sample’s target molecules such as spike (S) protein and glucose. We then test the compatibility of the pretreatment system with our previously designed SARS-CoV-2 and glucose diagnostic biosensing systems for detecting S protein and glucose in subject saliva. Ultimately, the effectiveness of each filter in enhancing biomarker sensitivity is assessed. The results show that a 20 mg nylon wool (NW) filter shows an 80% change in viscosity reduction with only a 6% reduction in protein content, making it an appropriate filter for the salivary S protein diagnostic system. Meanwhile, a 30 mg cotton wool (CW) filter is identified as the optimal choice for salivary glucose detection, achieving a 90% change in viscosity reduction and a 60.7% reduction in protein content with a minimal 4.3% reduction in glucose content. The NW pretreatment filtration significantly improves the limit of detection (LOD) for salivary S protein detection by five times (from 0.5 nM to 0.1 nM) and it reduces the relative standard deviation (RSD) two times compared to unfiltered saliva. Conversely, the CW filter used for salivary glucose detection demonstrated improved linearity with an R^2^ of 0.99 and a sensitivity of 36.6 μA/mM·cm^2^, over twice as high as unfiltered saliva. This unique filtration process can be extended to any POC diagnostic system and optimized for any biomarker detection, making electrochemical POC diagnostics more viable in the current market.

## 1. Introduction

POC testing (POCT) is medical diagnostic testing near the patient and outside the clinical laboratory but still in a medically supervised setting [1]. The main goal of POC diagnostics is to provide a rapid, convenient, and accurate testing system to the patient [2]. Self-monitoring blood glucometers, urine pregnancy testing, and COVID-19 testing kits are the most common POC devices. Among the various techniques used in POC testing, electrochemical biosensors have gained much attention recently due to their inherent advantages [3], comprising rapidity, sensitivity, specificity, and versatility, which set them apart from other detection methods [4,5,6]. Moreover, salivary electrochemical diagnostics is a growing field in POC technology and can be a superior alternative to blood, nasal fluid, and urine as a diagnostic medium due to its non-invasiveness and diverse biomolecule content [7,8,9,10].

Saliva is crucial in maintaining oral health and is a clinically informative biofluid that can reflect the body’s overall health [11] and is widely known as suitable for specific disease biomarker detection. It is 99% water [12] but also comprises electrolytes [13], mucus [14], enzymes [15], and a wide range of disease biomarkers. It has proven effective in electrochemical biosensing of various diseases such as HIV [16], hepatitis A [17], B [18], and C viruses [19], the Zika virus (ZIKV) [20,21], severe acute respiratory syndrome coronavirus (SARS-CoV) [22], coronavirus disease 2019 (COVID-19) [23,24], oral cancer [25], breast cancer [26], pancreatic cancer [27], lung cancer [28], cardiovascular disease [29], and diabetes [30,31]. Additionally, the simplified collection process allows the use of salivary-based measurements at home or in a laboratory without the need for trained personnel to oversee the process [32,33,34].

Despite the immense potential of saliva, detecting specific biomarkers in saliva can come with various challenges. Firstly, the concentrations of most biomarkers in saliva are significantly lower than their corresponding concentrations in blood, which raises the bar for the sensitivity of diagnostic devices that aim to use it [13,35,36]. Secondly, while electrochemical methods offer advantages such as simplicity, speed, and cost-effectiveness, they have not yet been widely applied to actual biological samples. A key factor limiting the effectiveness of electrochemical methods for use with saliva is the interference caused by numerous other proteins, biomarkers, and foreign particulates compromising the test’s selectivity and specificity [37]. These typically include food residues, large molecules, and/or high molecular weight (HMWT) protein chains, particularly those larger and more concentrated than the analyte to be detected. These large molecules can obstruct the diffusion of the analyte toward the sensor surface and block binding sites during electrochemical measurement, resulting in reduced sensor sensitivity. Of these large molecules, the family of mucin proteins is one of the most abundant in saliva and is a primary interferent in salivary biomarker measurements [38]. The brush-like structure of mucin along with its negatively charged backbone makes saliva samples more viscous [39,40]. Removing mucin leads to decreased viscosity of salivary samples where viscosity correlates with mucin concentration such that lower viscosity samples provide better sensor performance. Thus, the pretreatment of saliva is a pivotal step in utilizing saliva for electrochemical and other sensing systems.

In this research, we address the abovementioned issues by removing the most significant interferents, isolating the target biomarkers, and reducing viscosity (mucin). This objective is achieved by employing specific filter materials tailored to each relevant biomarker. The proposed filtration system is a critical link between laboratory and POC systems for saliva-based detection technology. This study investigates various filter materials for saliva treatment to facilitate the detection of SARS-CoV-19 S protein and glucose in real samples. Finally, to assess the effectiveness of our saliva pretreatment system, filtered saliva is tested with our designed biosensor for each biomarker and the detection sensitivity is compared with that of unfiltered saliva. 

## 2. Experimental Procedure

### 2.1. Material and Method

SARS-CoV-2 monoclonal S antibody and S protein of SARS-CoV-2 were purchased from Genscript. Gold nanoparticles (GNp, 10 nm diameter), PBS (0.1 mol/L, pH 7.4), thionine acetate salt (TH), tergitol 15-s-15 surfactant, chitosan (CS), GOx (type II lyophilized powder with at least 17,300 units/g solid, enzyme commission (EC) 1.1.3.4 enzyme from Aspergillus Niger), and acetate buffer solution (pH 4.65) were purchased from Sigma Aldrich. Semiconducting COOH functionalized single-walled carbon nanotube suspension (SWCNT-COOH, diameter: 1–2 nm, length: 2–5 nm) was purchased from Cheaptube Company, polyvinylidene fluoride (PVDF), polytetrafluoroethylene (Teflon) (PTFE), mixed cellulose ester (MCE), anotop (alumina-based), and nylon (NY) syringe filters were purchased from the Sigma company. Nylon wool (NW), glass wool (GW), cotton wool (CW), a BCA assay kit, and a COVID-19 S protein ELISA kit were purchased from Fisher Scientific. Cotton swabs were purchased from Oasis Company. The glucose assay kit was purchased from Abcam company. The viscosity test was performed using the microVISCTM device from “Rheosense Inc.” (San Ramon, CA, USA). The pressure gauge was purchased from the Life Science company.

### 2.2. Saliva Sample Collection Protocols

**Figure 1:**
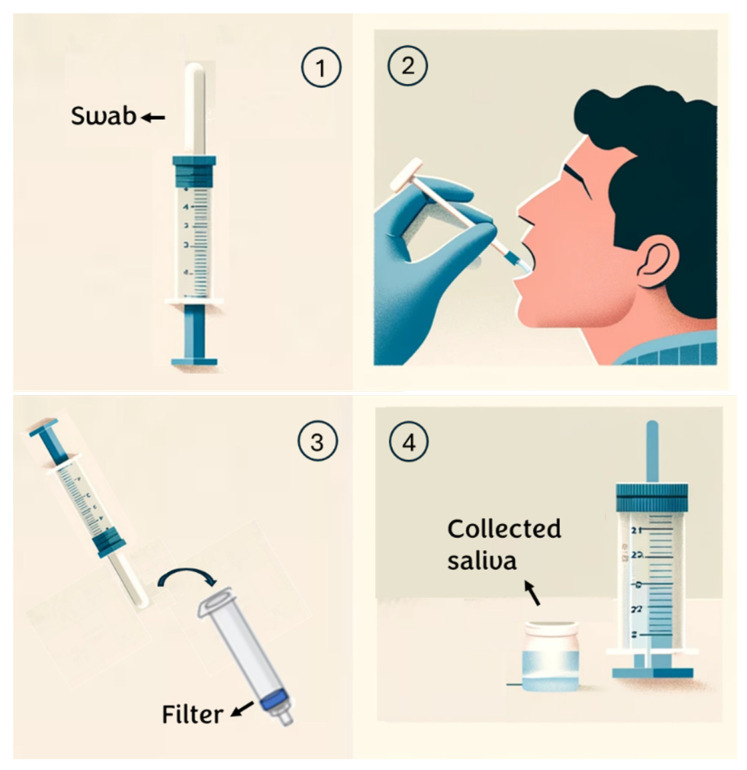
Saliva collection steps.

For this research, healthy volunteers aged between 20 and 30 years are selected. To ensure the proper execution of the test on these subjects, the following procedure is provided (Figure 1):

1. Individuals are instructed to rinse their mouths with water 15 min before saliva collection;
2. An absorbent pad is positioned inside the mouth and participants are instructed to chew on it until it is saturated with saliva (typically taking 30 to 60 s);
3. Subsequently, the saliva collector is inserted into a syringe containing a specific membrane/depth filter and the plunger is squeezed to extract the saliva from the absorbent pad;
4. The sample is transferred into a sterile tube for subsequent analysis. (see Appendix A)

### 2.3. Viscosity and BCA Measurement

**Figure 2:**
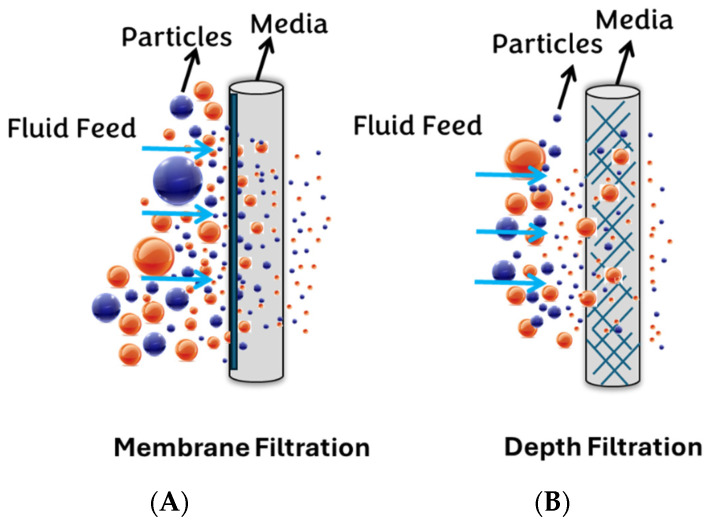
Depicts two categories of filtration: (**A**) membrane filtration and (**B**) depth filtration.

Generally, filter media can be classified into two major groups: surface (membrane) filters and depth filters (Figure 2). This study selects PVDF and NY, PTFE, MCE, and Anotop (Alumina-based) filters as the surface membrane syringe filters, while GW, NW, and CW are chosen as the depth filters. 

The performance of both types of filters (membrane and depth) is assessed by conducting two primary tests. First, the viscosity of saliva is measured as an indication of mucin concentration by a microVISCTM viscometer (Supplementary Materials). To assess the effectiveness of a filter in reducing mucin levels in saliva, the viscosity of the samples is determined using viscosity measurements. It should be noted that the viscosity tests were conducted under similar environmental conditions (Temperature 22 °C and humidity 20%) to ensure that the variation in viscosity is minimal. Also, the experiments were repeated three times and, based on the obtained results, the standard error of viscosity was lower than 2%, indicating the stability and accuracy of the results.

Equation (1) is used to calculate the percent of viscosity reduction change parameter (ΔM), which measures the degree to which a filter returns the viscosity of filtered sample to that of an ideal buffer solution that has no protein content.

(1) $$\Delta M=\frac{VU-VF}{VU-VB}\times 100$$

where VU is the viscosity of unfiltered saliva, VF  is the viscosity of filtered saliva, and VB  is the viscosity of buffer solution.

To evaluate the filtration efficiency concerning other proteins, a Bicinchoninic Acid Assay (BCA) kit is employed as a standardized method for quantifying the total protein concentration present in filtered and unfiltered saliva samples.

### 2.4. Biosensor Fabrication Procedure

#### 2.4.1. SARS-CoV-2 Biosensor 

**Figure 3:**
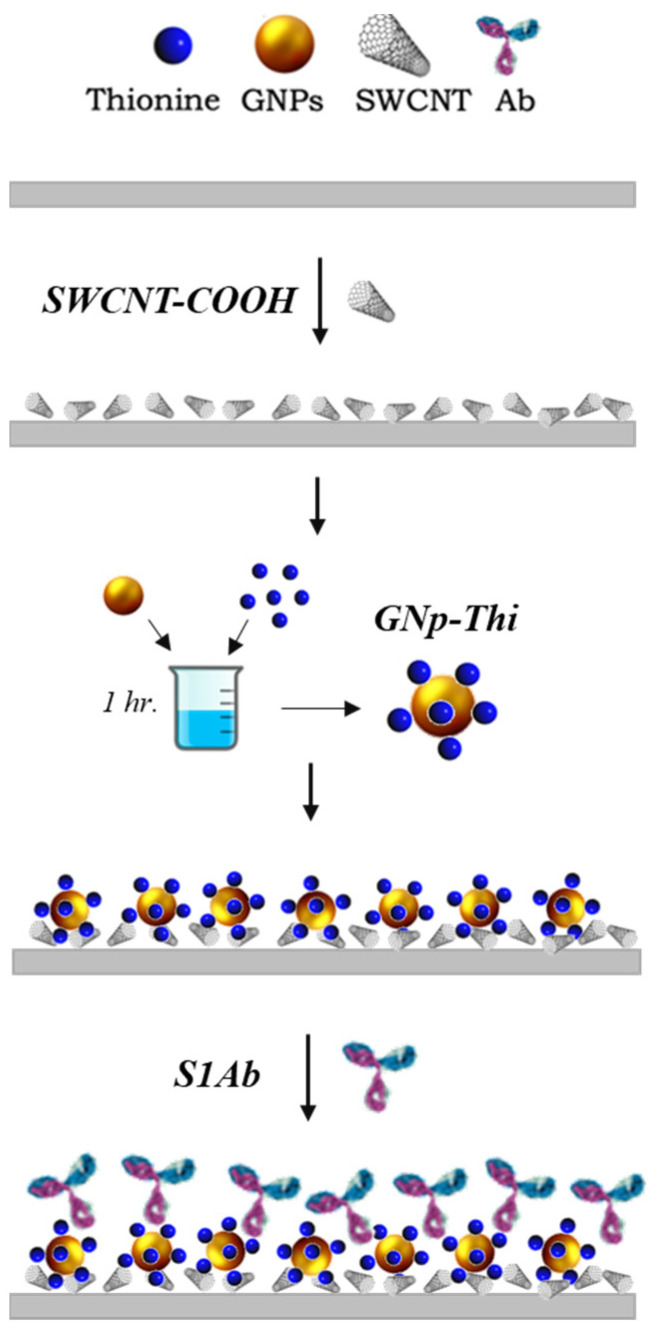
Fabrication procedure of the SARS-CoV-2 biosensor chemistry design.

In brief, 1 μL of the SWCNT-COOH solution is drop-cast on the WE surface and dried in a desiccator for 20 min. Then, TH solution is dissolved in water and incubated with GNp (10 nm) at room temperature for 20 min. The volume ratio of 1:1.5 for GNp and TH was mixed for 1 h at room temperature in dark conditions. Then, 1.5 µL of the mixture GNp+TH is dropped onto the surface. Afterward, 1.5 μL of this GNp+TH solution is then dropped on the working electrode (WE) and the sensors are left to dry in the desiccator. Lastly, 2 μL of anti-S protein antibody in 0.1 M PBS is dropped on the GNp+TH/SWCNT-COOH. After functionalization, the modified sensor is placed in a vacuum-sealed dark container and stored at 4 °C for later use. The fabrication procedure details are in the following work [41] and schematically depicted in Figure 3.

#### 2.4.2. Glucose Biosensor 

**Figure 4:**
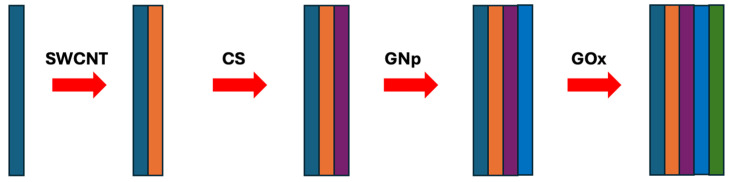
The fabrication procedure of the glucose biosensor chemistry design.

In brief, 10 μL of SWNT-COOH suspension is cast onto each sensor. Then, 10 μL of 2 mg/mL CS, 10 μL of GNp (10 nm), and 10 μL of 1 mg/mL GOx are cast onto the WE sequentially to form the first (SWCNT-COOH/CS/GNp/GOx) multi-layer film with drying steps in between, where each layer takes 20 min to complete. Subsequently, two more multi-layer films are cast on top, after which the sensors are dried in the desiccator for 1 h without a washing step. They are then packed in gel boxes (Gel-Pak, Hayward, CA, USA) and sealed in vacuum bags using a vacuum packaging machine (VACmaster pro110, Overland Park, KS, USA) and stored at 4 °C for later use. The fabrication procedure details are discussed in detail in [42] and shown schematically in Figure 4. 

### 2.5. Electrochemical Testing Procedure and Techniques

To evaluate the effect of saliva pretreatment on biosensor sensitivity, the selected biomarker is added to the subject’s saliva samples, which are then filtered using a selected filter. This is followed by square wave voltammetry (SWV) for the S protein sensors or amperometry for the glucose sensors and the results are compared with those from unfiltered saliva samples. The measurement process begins with connecting the sensor to a PalmSens4 potentiostat. Then, 20 μL of the spiked sample is dispensed onto the electrode area to cover all three sensor electrodes. After the incubation period, the corresponding electrochemical test is performed.

During SWV, the applied potential range is −0.2 V to 0.4 V with an E_step_ of 5 mV and an E_amplitude_ of 0.05 V. The current is recorded for the baseline (blank) and analyte solution and a Δi is calculated to analyze the level of binding and detection through Equation (2).

(2) $$\Delta i=i_{pB}-i_{pS}$$

where *i_pB_* is the peak current of the baseline solution and *i_pS_* is the peak current of the analyte solution such as in this work [41]. During amperometry, −0.5 V potential is applied to the functionalized sensor and the resulting current is integrated from 18–21 s and analyzed for each concentration as in this work [42]. All experiments are performed in triplicate to ensure the reproducibility of the results and the *LOD* value in all the electrochemical measurements is calculated using Equation (3).

(3) $$LOD=\frac{\left(3.3\times \sigma \right)}{S}$$

Here, σ is the average standard deviation of blank sensor response and S is the slope of the linear calibration plot. 

## 3. Results and Discussion

### 3.1. Filter Assessment and Selection

#### 3.1.1. Syringe Membrane Filters

Wettability characteristics are a crucial consideration when choosing a filter for a particular application. Hydrophobic membranes may only be suitable for water-based samples as the force required to push sample through becomes unreasonably high. Therefore, hydrophilic filter types are appropriate for use and are selected for saliva pretreatment. Table 1 summarizes the syringe membrane filter’s viscosity and total protein test results of hydrophilic membranes. The overall results indicate that hydrophilic PVDF achieved the highest efficacy in reducing saliva viscosity, with reductions of approximately 93% of saliva viscosity. Also, hydrophilic PES and hydrophilic PVDF demonstrated the highest performance regarding total protein removal with reduction of 51% and 50%. 

As molecules like SARS-CoV-2 S protein and glucose have vastly different characteristics with respect to size and weight, it follows that different filter types may be needed for each.

Since glucose is a small molecule, a more significant reduction in protein content becomes the preferable option for this biomarker as removing all protein would remove most of the interferents. Among all the filters, PVDF shows excellent performance in reducing viscosity and total protein content and is selected as the best membrane filter for salivary glucose detection.

Although membrane filters effectively reduce viscosity and protein content, none are suitable for protein biomarkers such as S proteins since they reduce about 50% of the protein from saliva. Therefore, there is a high likelihood that the S protein becomes trapped in the membrane filters. 

#### 3.1.2. Depth Filters 

Three depth filter types of varying weights are evaluated with respect to their viscosity and protein reduction to identify the most suitable depth filter for the saliva collection system. To identify an appropriate filter weight, the experiment started using 10 mg for each filter. It was found that this weight did not provide high confidence in fully covering the syringe. Consequently, the starting point was adjusted to 20 mg and the weight was incrementally increased. However, as additional weight was added, the results deviated from the target values in terms of percentage change in glucose and S protein levels. Thus, further weight additions were ceased and this weight range was established as the standard.

Additionally, to ascertain the percentage change in each biomarker concentration before and after filtration, both the SARS-CoV-2 S protein ELISA kit and the glucose assay kit were utilized. As depicted in Table 2, the results indicate that the NW filter, weighing 20 mg, showed the most favorable performance. It achieved around 83.2% reduction in viscosity, only a 6% reduction in protein content, and a minimal 3% change in S protein concentration. Therefore, the NW depth filter weighing 20 mg has been selected for use in the salivary S protein biosensor. Conversely, for glucose, the CW with a 30 mg weight exhibited the best performance, demonstrating a viscosity reduction parameter of 96% and a 58% reduction in protein content while causing only a minimal change in glucose content (2.3%).

#### 3.1.3. Water Breakthrough Pressure

Before evaluating the filter performance in biomarker sensitivity enhancement, the filter integrity should be assessed for a POCT diagnostic system. The water breakthrough pressure is an essential metric to understand for any filter to ensure that the required force for passing saliva samples through these membranes is reasonable for users. In this experiment, the water breakthrough pressure is defined as the minimum pressure required to force the sample through the selected membranes and is quantified to compare the breakthrough pressure of filters. This involves using a pressure gauge, a syringe, and filters, including PVDF, 30 mg CW, and 20 mg NW filters (as depicted in Figure S1 in Supplementary Materials). The water breakthrough pressure $P_{WB}$ and the corresponding required force applied for passing water and saliva through each filter are detailed in Table 3.

Considering the above results, the PVDF membrane requires an unmanageable force of 14.63 N to pass saliva through the filter, making it unsuitable for POC diagnostic detection. However, 30 mg CW and 20 mg NW remain the preferred options since they require less force from users to perform the test. In conclusion, Table 4 shows the optimal filtration system for the SARS-CoV-2 and glucose POC detection system.

### 3.2. Filter Sensitivity Assessment 

#### 3.2.1. Salivary Glucose Measurement 

**Figure 5:**
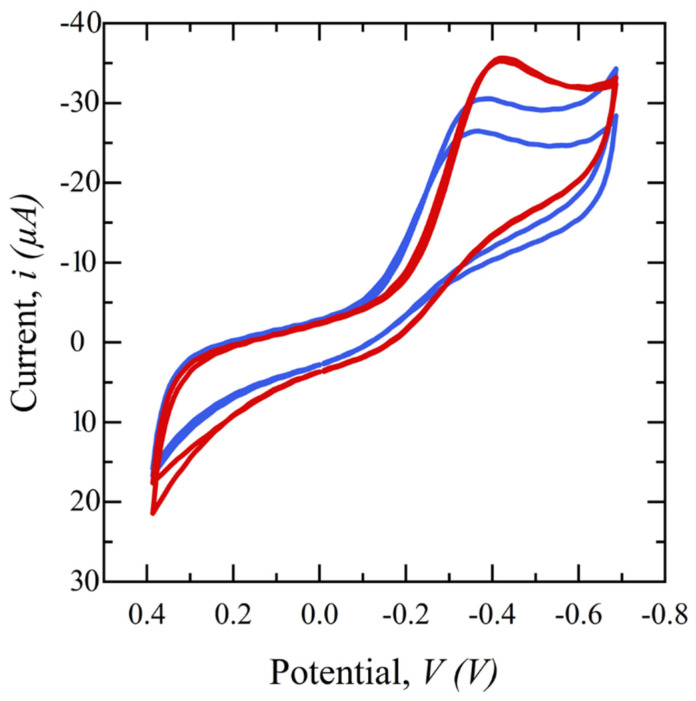
Cyclic voltammograms comparing filtered saliva (red) and unfiltered saliva (blue) in a potential range of −0.4 to 0.8 V, scan rates of 50 mV/s, and 1 ms interval.

To study the effect of saliva filtration on the electrochemical behavior of the sensor, cyclic voltammetry (CV) in a potential range of −0.8 to 0.4 V is performed on glucose electrochemical biosensors using CW-filtered and unfiltered saliva. Figure 5 shows the subsequent cyclic voltammograms. Both samples have the same spiked glucose concentration of 5 mg/dL but the redox reaction peak of the filtered saliva sample is sharper, higher, and more repeatable than that of the unfiltered saliva, demonstrating the superiority of the filtered sample. This is hypothesized to be because the presence of interferents can limit the diffusion of glucose from the bulk to the sensor surface and/or block enzyme binding sites on the electrode surface, preventing a glucose-GOx reaction.

**Figure 6:**
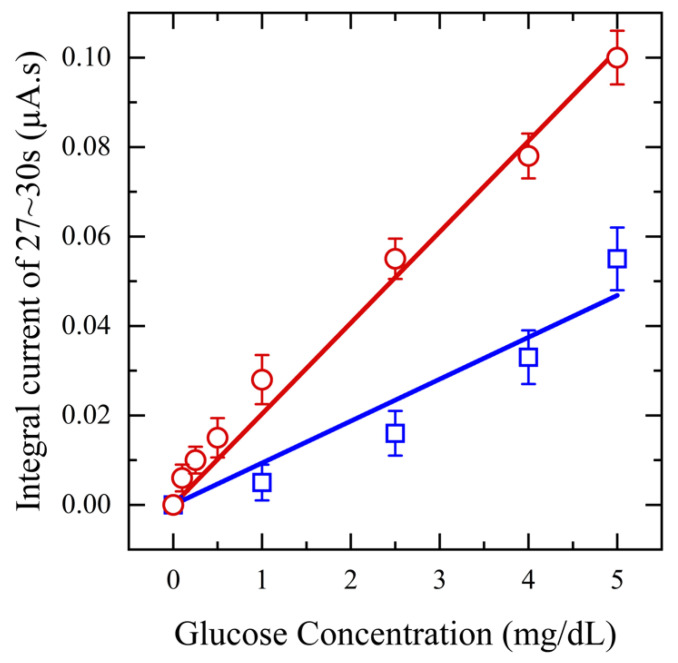
Amperometry for functionalized sensors with different concentrations of glucose from (0.1–5 mg/dL) in saliva (red) and unfiltered saliva (blue) at applied potential 0.5 V.

Next, to assess the impact of saliva pretreatment on glucose detection sensitivity, a range of glucose concentrations (0.1–5 mg/dL) is spiked into collected saliva samples, which are then filtered using a 20 mg CW filter. Amperometry measurements are performed at an applied potential of 0.5 V for 20 s and the resulting output current is integrated from 17–20 s, plotted against concentration, and compared with that of unfiltered saliva samples. These tests are performed in triplicate for each concentration. The results in Figure 6 clearly show that using filtered saliva enhances the range of biosensor detection from 1 mg/dL to 0.1 mg/dL. The current response increased with rising glucose concentration, demonstrating a linear relationship with a sensitivity of 36.6 μA/mM·cm^2^ and an R^2^ value of 0.99 for filtered saliva. In contrast, unfiltered saliva exhibited a lower sensitivity, 16.7 μA/mM·cm^2^, with an R^2^ value of 0.96. This decrease can be attributed to mucin and other proteins, which can hinder the diffusion of glucose molecules and obstruct the binding sites, consequently diminishing the sensor’s sensitivity. The RSD of less than 3% for the filtered saliva data points compared to unfiltered saliva, confirms enhanced repeatability.

#### 3.2.2. Salivary SASR-CoV-2 S Measurement

**Figure 7:**
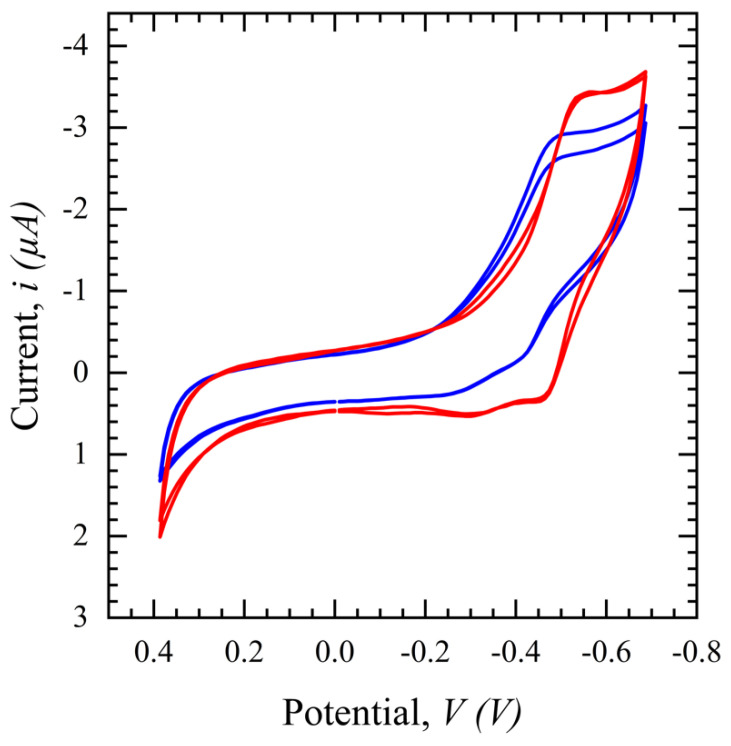
Cyclic voltammograms comparing filtered saliva (red) and unfiltered saliva (blue) in a potential range of 0.4 to −0.8 V, scan rates of 100 mV/s, and a 1 ms interval.

CV in a potential range of 0.4 to −0.8 V with a scan rate of 100 mV/s is performed for the SARS-CoV-2 S protein biosensor and the same phenomenon as seen with the glucose sensor in Figure 7 is observed in Figure 7. The CV peak for filtered saliva shows a higher current with more repeatability and less variation than unfiltered saliva. This demonstrates the impact of other interferents present in the solution, which are hypothesized to disrupt the path of ions necessary to sustain the TH embedded in the electrical double layer and/or land on the surface and block binding sites. In conclusion, the filtration process purifies the sample, resulting in less variation and a higher output signal for biomarker detection.

**Figure 8:**
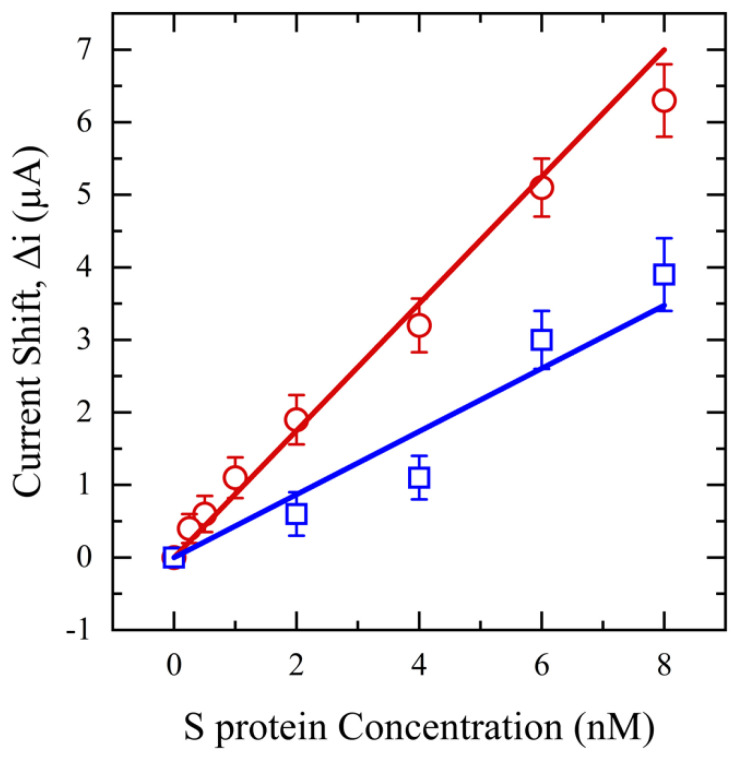
SWV for functionalized sensors with different concentrations of S protein (0.25–8 nM) in saliva (red) and unfiltered saliva (blue).

Next, SWV is performed in a range of 0–0.7 V for an S protein concentration range of 0.25–8 nM. These findings are shown in Figure 8 and highlight the influence of saliva pretreatment on sensor performance and S protein measurement. Remarkably, despite both filtered and unfiltered samples having the same concentration of S protein, the sensor can detect concentrations as low as 0.25 nM with an R^2^ of 0.99 and a limit of detection (LOD) of 0.1 nM for filtered saliva. However, the sensor’s detection capability is reduced to 2 nM for unfiltered saliva, with an LOD of 0.5 nM and an R^2^ of 0.96. This diminished sensitivity is again likely due to interfering substances in unfiltered saliva, which may impede the mobility of the S protein or block the active sites of the antibody on the electrode surface, thus preventing effective interaction between the S protein and the antibody. Additionally, the relative standard deviation for unfiltered saliva is >4%, at least two times higher than filtered saliva due to high molecular weight mucin and food debris deposition on the sensor surface, inducing variation and noise in the output signal.

## 4. Conclusions

In this study, the necessary criteria for pre-treatment of salivary glucose and S protein samples are established and various filters for saliva treatment are examined for each biomarker. The 30 mg NW filter is chosen for S protein samples, while the 20 mg CW filter is selected for glucose samples. To validate the efficacy of sample pre-treatment, the performance of saliva filtration was assessed using our developed biosensors for SARS-CoV-2 and glucose. The results indicate that the saliva filtration pretreatment idea has great potential to enhance the sensitivity of saliva-based electrochemical POC/OTC biosensors. 

The NW pretreatment filtration significantly improved the limit of detection (LOD) for salivary S protein detection by five times (from 0.5 nM to 0.1 nM) and RSD by two times compared to unfiltered saliva. Conversely, the CW used for salivary glucose detection demonstrated improved linearity with an R^2^ of 0.99 and a sensitivity of 36.6 μA/mM·cm^2^, around twice as high as unfiltered saliva.

Consequently, the pre-treatment system for saliva is versatile and can be widely applied to a range of electrochemical biosensors. By customizing the filter’s type, size, and weight, it can be fine-tuned for detecting different biomarkers, enhancing both sensitivity and reliability. Such flexibility enhances the practicality of electrochemical biosensors as an effective POC device.

## 5. Future Work

Our future work will focus on conducting clinical tests using actual saliva samples from patients that contain viruses and further optimizing the filter for the diagnostic detection of whole viruses. We will consider studying the effect of pore size on the efficacy of saliva treatment and biosensor performance, as the size of the target particles plays a crucial role in the filtration system.

## Figures and Tables

**Table 1:** Selected membrane filters with their corresponding viscosity, reduction, and protein reduction test results.

Sample	Hydrophobicity	Average Viscosity (mPa·s)	Δ*M* (%)	Protein Concentration (µg/mL)	Total Protein Reduction (%)
Buffer		1.04			
Unfiltered saliva		1.19		3001.21	
PVDF filtered saliva	Hydrophilic	1.05	93	1500.60	50
Anotop filtered saliva	Hydrophilic	1.1	60	1680.67	44
NY filtered saliva	Hydrophilic	1.08	73	1710.68	43
MCE filtered saliva	Hydrophilic	1.06	86	1470.59	51

**Table 2:** Viscosity and protein reduction test results of filtered saliva using three wool filter types with three different weights.

Filter	Weight (mg)	Δ*M* (%)	Total Protein Reduction (%)	Change in Glucose Content (%)	Change in S protein Content (%)
NW	20	83.2	6	10	3
NW	30	85	10.7	17.3	14.3
NW	40	88.6	16.1	13.5	17.4
GW	20	75	14.4	0	13.1
GW	30	88.8	15.6	7.1	17.4
GW	40	87	21	9.4	24.5
CW	20	92	47.7	4.2	40
CW	30	96	58.7	2.3	50.2
CW	40	90	40.8	11.8	46

**Table 3:** Water breakthrough pressure test results for selected hydrophilic filters.

Sample	Filter	Pressure (×100 kPa)	Force (N) (≈0.22481 lbs.)
Water	PVDF	1	13.3
Water	CW	0.4	5.32
Water	NW	0.3	3.99
Saliva	PVDF	1.1	14.63
Saliva	CW	0.4	5.32
Saliva	NW	0.6	7.98

**Table 4:** Optimal filtration system for salivary SARS-CoV-2 and glucose detection.

Biomarker	SARS-CoV-2 S	Glucose
Filter	NW	CW
Weight (mg)	20	30
Hydrophobicity	Hydrophilic	Hydrophilic
ΔM (%)	88.7	90
Protein Reduction (%)	6	60.7
Change in S Protein Content (%)	3	50.2
Change in Glucose content (%)	10	2.3
Force (N)	7.98	5.32

## Data Availability

All data have been reported in the manuscript.

## Supplementary Materials

The following supporting information can be downloaded at: https://www.mdpi.com/article/10.3390/diagnostics14111088/s1, Figure S1: The viscometer measures saliva samples’ viscosity (Rheosense Inc, San Roman, CA, USA); Figure S2: The water breakthrough test setup.

## Appendix A

Since our lab is BSL-2-certified and cannot use patient saliva containing whole viruses, we are limited to collecting saliva from healthy patients and spiking it with the biomarker. Based on the future work outlined, the project’s next phase will involve using saliva samples from infected patients and further optimizing the saliva filter pretreatment since the whole virus is 10^4^ times larger than the Spike protein.

## References

[1] Florkowski C., Don-Wauchope A., Gimenez N., Rodriguez-Capote K., Wils J., Zemlin A. (2017). Point-of-care testing (POCT) and evidence-based laboratory medicine (EBLM)—Does it leverage any advantage in clinical decision making?. Crit. Rev. Clin. Lab. Sci..

[2] Esfahani I.C., Sun H. (2023). A droplet-based micropillar-enhanced acoustic wave (μPAW) device for viscosity measurement. Sens. Actuators A Phys..

[3] Da Silva E.T.S.G., Souto D.E.P., Barragan J.T.C., Giarola J.d.F., de Moraes A.C.M., Kubota L.T. (2017). Electrochemical biosensors in point-of-care devices: Recent advances and future trends. ChemElectroChem.

[4] Wan Y., Su Y., Zhu X., Liu G., Fan C. (2013). Development of electrochemical immunosensors towards point of care diagnostics. Biosens. Bioelectron..

[5] Wang J. (2006). Electrochemical biosensors: Towards point-of-care cancer diagnostics. Biosens. Bioelectron..

[6] Farsaei Vahid N., Marvi M.R., Naimi-Jamal M.R., Naghib S.M., Ghaffarinejad A. (2018). Effect of surfactant type on buckypaper electrochemical performance. Micro Nano Lett..

[7] Pittman T.W., Decsi D.B., Punyadeera C., Henry C.S. (2023). Saliva-based microfluidic point-of-care diagnostic. Theranostics.

[8] Mani V., Beduk T., Khushaim W., Ceylan A.E., Timur S., Wolfbeis O.S., Salama K.N. (2021). Electrochemical sensors targeting salivary biomarkers: A comprehensive review. TrAC Trends Anal. Chem..

[9] Dabiri D., Dehghan Banadaki M., Bazargan V., Schaap A. (2023). Numerical investigation of moving gel wall formation in a Y-shaped microchannel. SN Appl. Sci..

[10] Akhavani S.A., Jueckstock J., Su J., Kapravelos A., Kirda E., Lu L. (2021). Browserprint: An Analysis of the Impact of Browser Features on Fingerprintability and Web Privacy.

[11] Lamkin M.S., Oppenheim F.G. (1993). Structural features of salivary function. Crit. Rev. Oral Biol. Med..

[12] Humphrey S.P., Williamson R.T. (2001). A review of saliva: Normal composition, flow, and function. J. Prosthet. Dent..

[13] De Almeida P.D.V., Gregio A.M., Machado M.A., De Lima A.A., Azevedo L.R. (2008). Saliva composition and functions: A comprehensive review. J. Contemp. Dent. Pract..

[14] Wotman S., Mandel I.D., Thompson R.H., Laragh J.H. (1967). Salivary electrolytes, urea nitrogen, uric acid and salt taste thresholds in hypertension. J. Oral Ther. Pharmacol..

[15] Chauncey H.H., Lionetti F., Winer R.A., Lisanti V.F. (1954). Enzymes of human saliva: I. The determination, distribution, and origin of whole saliva enzymes. J. Dent. Res..

[16] Mortimer P.P., Parry J.V. (1994). Detection of antibody to HIV in saliva: A brief review. Clin. Diagn. Virol..

[17] Oba I.T., Spina A.M.M., Saraceni C.P., Lemos M.F., Senhoras R.d.C.F.A., Moreira R.C., Granato C.F.H. (2000). Detection of hepatitis A antibodies by ELISA using saliva as clinical samples. Rev. Inst. Med. Trop. São Paulo.

[18] Arora G., Sheikh S., Pallagatti S., Singh B., Singh V.A., Singh R. (2012). Saliva as a tool in the detection of hepatitis B surface antigen in patients. Compend. Contin. Educ. Dent..

[19] González V., Martró E., Folch C., Esteve A., Matas L., Montoliu A., Grífols J.R., Bolao F., Tural C., Muga R. (2008). Detection of hepatitis C virus antibodies in oral fluid specimens for prevalence studies. Eur. J. Clin. Microbiol. Infect. Dis..

[20] Khurshid Z., Zafar M., Khan E., Mali M., Latif M. (2019). Human saliva can be a diagnostic tool for Zika virus detection. J. Infect. Public Health.

[21] Esfahani I.C., Ji S., Sun H. (2023). A Drop-on-Micropillars (DOM) based Acoustic Wave Viscometer for High Viscosity Liquid Measurement. IEEE Sens. J..

[22] Wang W.-K., Chen S.-Y., Liu I.J., Chen Y.-C., Chen H.-L., Yang C.-F., Chen P.-J., Yeh S.-H., Kao C.-L., Huang L.-M. (2004). Detection of SARS-associated coronavirus in throat wash and saliva in early diagnosis. Emerg. Infect. Dis..

[23] Kim Y.-I., Kim S.-G., Kim S.-M., Kim E.-H., Park S.-J., Yu K.-M., Chang J.-H., Kim E.J., Lee S., Casel M.A.B. (2020). Infection and rapid transmission of SARS-CoV-2 in ferrets. Cell Host Microbe.

[24] Nazarian S., Farsaeivahid N., Grenier C., Yunqing D.U., Wang M.L. (2021). Rapid Electrochemical Point-of-Care COVID-19 Detection in Human Saliva. Google Patents.

[25] Jasim H., Carlsson A., Hedenberg-Magnusson B., Ghafouri B., Ernberg M. (2018). Saliva as a medium to detect and measure biomarkers related to pain. Sci. Rep..

[26] Streckfus C.F., Bigler L.R., Zwick M. (2006). The use of surface-enhanced laser desorption/ionization time-of-flight mass spectrometry to detect putative breast cancer markers in saliva: A feasibility study. J. Oral Pathol. Med..

[27] Asai Y., Itoi T., Sugimoto M., Sofuni A., Tsuchiya T., Tanaka R., Tonozuka R., Honjo M., Mukai S., Fujita M. (2018). Elevated polyamines in saliva of pancreatic cancer. Cancers.

[28] Qian K., Wang Y., Hua L., Chen A., Zhang Y. (2018). New method of lung cancer detection by saliva test using surface-enhanced Raman spectroscopy. Thorac. Cancer.

[29] Basu A., Zinger T., Inglima K., Woo K.-M., Atie O., Yurasits L., See B., Aguero-Rosenfeld M.E. (2020). Performance of Abbott ID Now COVID-19 rapid nucleic acid amplification test using nasopharyngeal swabs transported in viral transport media and dry nasal swabs in a New York City academic institution. J. Clin. Microbiol..

[30] Jurysta C., Bulur N., Oguzhan B., Satman I., Yilmaz T.M., Malaisse W.J., Sener A. (2009). Salivary glucose concentration and excretion in normal and diabetic subjects. BioMed Res. Int..

[31] Vahid N.F., Marvi M.R., Naimi-Jamal M.R., Naghib S.M., Ghaffarinejad A. (2019). X-Fe_2_O_4_-buckypaper-chitosan nanocomposites for nonenzymatic electrochemical glucose biosensing. Anal. Bioanal. Electrochem..

[32] Williamson S., Munro C., Pickler R., Grap M.J., Elswick R.K. (2012). Comparison of biomarkers in blood and saliva in healthy adults. Nurs. Res. Pract..

[33] Hofman L.F. (2001). Human saliva as a diagnostic specimen. J. Nutr..

[34] Chojnowska S., Baran T., Wilińska I., Sienicka P., Cabaj-Wiater I., Knaś M. (2018). Human saliva as a diagnostic material. Adv. Med. Sci..

[35] Yoshizawa J.M., Schafer C.A., Schafer J.J., Farrell J.J., Paster B.J., Wong D.T.W. (2013). Salivary biomarkers: Toward future clinical and diagnostic utilities. Clin. Microbiol. Rev..

[36] Shirtcliff E.A., Granger D.A., Schwartz E., Curran M.J. (2001). Use of salivary biomarkers in biobehavioral research: Cotton-based sample collection methods can interfere with salivary immunoassay results. Psychoneuroendocrinology.

[37] Ngamchuea K., Chaisiwamongkhol K., Batchelor-McAuley C., Compton R.G. (2018). Chemical analysis in saliva and the search for salivary biomarkers—A tutorial review. Analyst.

[38] Carpenter G.H. (2013). The secretion, components, and properties of saliva. Annu. Rev. Food Sci. Technol..

[39] Acquier A.B., Pita A.K.D.C., Busch L., Sánchez G.A. (2015). Comparison of salivary levels of mucin and amylase and their relation with clinical parameters obtained from patients with aggressive and chronic periodontal disease. J. Appl. Oral Sci..

[40] Esfahani I.C., Ji S., Alamgir Tehrani N., Sun H. (2023). An ultrasensitive micropillar-enabled acoustic wave (μPAW) microdevice for real-time viscosity measurement. Microsyst. Technol..

[41] Farsaeivahid N., Grenier C., Nazarian S., Wang M.L. (2022). A rapid label-free disposable electrochemical salivary point-of-care sensor for SARS-CoV-2 detection and quantification. Sensors.

[42] Du Y., Zhang W., Wang M.L. (2016). Sensing of salivary glucose using nano-structured biosensors. Biosensors.

