# Supplementary material for: Filtered Saliva for Rapid and Accurate Analyte Detection for POC Diagnostics
Source: Diagnostics (Basel). 2024 May 24;14(11):1088. doi: 10.3390/diagnostics14111088 (PMC11171550; doi:10.3390/diagnostics14111088)
Supplement: Supplementary file 1 [file diagnostics-14-01088-s001.zip › diagnostics-2983435-supplementary.pdf]

Supplementary material

Filtered Saliva for Rapid and Accurate Analyte Detection for POC Diagnostics.

Nadia Farsaeivahid [1], Christian Grenier[1], Ming L. Wang[2,] *

1. Interdisciplinary Engineering Program, College of Engineering, Northeastern University, Boston, MA 02115, USA

2. Civil and Environmental Engineering Department, Northeastern University, Boston, MA 02115, USA

* Corresponding author: mi.wang@northeastern.edu

1- Viscosity Measurement

Viscosity measurement was performed using a microVISCTM viscometer (RheoSense, CA), as shown in Figure S1. In total, 200 uL of sample solution was used to measure the viscosity. The viscosity measurement was performed at a shear rate of 100 to 10000 1/s, a temperature of 22 ^{o}C, and a humidity of 20 %.

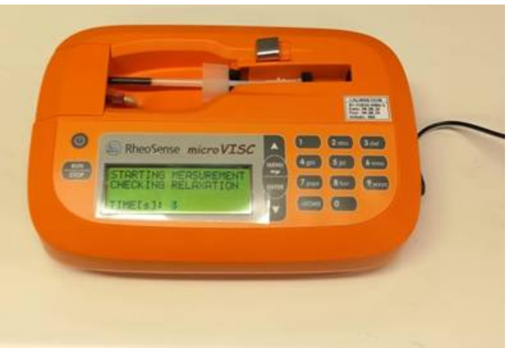

Figure S1. The viscometer measures saliva samples' viscosity (Rheosense Inc, San Roman, CA).

2- Water Breakthrough Pressure

Water breakthrough pressure was performed using a syringe, pressure gauge, and sample filter (Figure S2). To apply uniform pressure to the syringe, we utilized a microfluidic pump. The average volume of saliva collected per person using the same absorbent pad was around 200 μL. The target was to pass all 200 μL through the filter within 1 second. Accordingly, the infusion rate of the pump was set to meet this requirement. Subsequently, the average pressure was measured and reported.

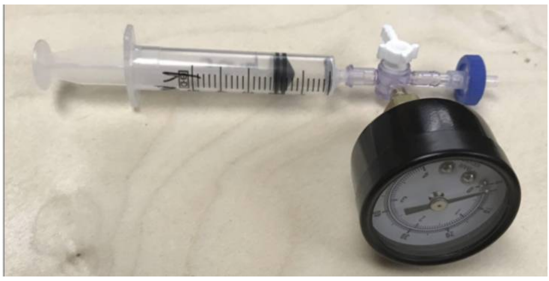

Figure S2. The water breakthrough test setup.
